# HLAIImaster: a deep learning method with adaptive domain knowledge predicts HLA II neoepitope immunogenic responses

**DOI:** 10.1093/bib/bbae302

**Published:** 2024-06-26

**Authors:** Qiang Yang, Long Xu, Weihe Dong, Xiaokun Li, Kuanquan Wang, Suyu Dong, Xianyu Zhang, Tiansong Yang, Feng Jiang, Bin Zhang, Gongning Luo, Xin Gao, Guohua Wang

**Affiliations:** School of Medicine and Health, Harbin Institute of Technology, Yikuang Street, Harbin 150000, China; School of Computer Science and Technology, Harbin Institute of Technology, West Dazhi Street, Harbin 150001, China; College of Computer and Control Engineering, Northeast Forestry University, Hexing Road, Harbin 150004, China; School of Computer Science and Technology, Harbin Institute of Technology, West Dazhi Street, Harbin 150001, China; School of Computer Science and Technology, Heilongjiang University, Xuefu Road, Harbin 150080, China; Postdoctoral Program of Heilongjiang Hengxun Technology Co., Ltd., Xuefu Road, Harbin 150090, China; Shandong Hengxun Technology Co., Ltd., Miaoling Road, Qingdao 266100, China; School of Computer Science and Technology, Harbin Institute of Technology, West Dazhi Street, Harbin 150001, China; College of Computer and Control Engineering, Northeast Forestry University, Hexing Road, Harbin 150004, China; Department of Breast Surgery, Harbin Medical University Cancer Hospital, Haping Road, Harbin 150081, China; Department of Rehabilitation, The First Affiliated Hospital of Heilongjiang University of Traditional Chinese Medicine, and Traditional Chinese Medicine Informatics Key Laboratory of Heilongjiang Province, Heping Road, Harbin 150040, China; School of Medicine and Health, Harbin Institute of Technology, Yikuang Street, Harbin 150000, China; Computer, Electrical and Mathematical Sciences & Engineering Division, King Abdullah University of Science and Technology, 4700 KAUST, Thuwal 23955, Saudi Arabia; Computer, Electrical and Mathematical Sciences & Engineering Division, King Abdullah University of Science and Technology, 4700 KAUST, Thuwal 23955, Saudi Arabia; Computer, Electrical and Mathematical Sciences & Engineering Division, King Abdullah University of Science and Technology, 4700 KAUST, Thuwal 23955, Saudi Arabia; College of Computer and Control Engineering, Northeast Forestry University, Hexing Road, Harbin 150004, China

**Keywords:** immunogenicity, tumor-specific antigen presentation, attention network, domain adaption, mass spectrometry data, HLA II allele

## Abstract

While significant strides have been made in predicting neoepitopes that trigger autologous CD4+ T cell responses, accurately identifying the antigen presentation by human leukocyte antigen (HLA) class II molecules remains a challenge. This identification is critical for developing vaccines and cancer immunotherapies. Current prediction methods are limited, primarily due to a lack of high-quality training epitope datasets and algorithmic constraints. To predict the exogenous HLA class II-restricted peptides across most of the human population, we utilized the mass spectrometry data to profile >223 000 eluted ligands over HLA-DR, -DQ, and -DP alleles. Here, by integrating these data with peptide processing and gene expression, we introduce HLAIImaster, an attention-based deep learning framework with adaptive domain knowledge for predicting neoepitope immunogenicity. Leveraging diverse biological characteristics and our enhanced deep learning framework, HLAIImaster is significantly improved against existing tools in terms of positive predictive value across various neoantigen studies. Robust domain knowledge learning accurately identifies neoepitope immunogenicity, bridging the gap between neoantigen biology and the clinical setting and paving the way for future neoantigen-based therapies to provide greater clinical benefit. In summary, we present a comprehensive exploitation of the immunogenic neoepitope repertoire of cancers, facilitating the effective development of “just-in-time” personalized vaccines.

## Introduction

Somatic mutations in cancer can generate tumor-specific neoantigens [1, 2]. These peptide products cleaved by protease are presented as neoepitopes bound to major histocompatibility complex class II (MHC-II) on the surface of professional antigen-presenting cells (APCs), such as B cells and dendritic cells [3–5]. These APCs primarily rely on human leukocyte antigen (HLA)-II molecules for antigen presentation on three loci on chromosome 6 (HLA-DR, -DQ, and DP), which encode corresponding heterodimeric proteins that composed of alpha and beta chains [6]. In tumors or infections, neoepitopes recognized by T cell receptors on CD4+ T cells can elicit strong immunoreactivity [7, 8], which is crucial for cancer immunotherapies, e.g. adoptive T cell transfer engineering and immune checkpoint blockade [9–11]. However, such T cell recognition of exogenous peptide-MHC (pMHC) II complex is challenging due to the various lengths of presented peptides and the high polymorphism of HLA alleles [12]. This makes it difficult to identify the core binding regions of HLA-II ligands, especially in datasets obtained from liquid chromatography-tandem mass spectrometry [13], as the ligands from the same patient are eluted from multiple HLA-II alleles.

In the field of cancer, deep learning-based methods are increasingly developed in next-generation sequencing (NGS) of tumors to explore cancer neoantigens and their immunogenic response, which arise from tumor-specific somatic mutations [14, 15]. These tools facilitate rapid and accurate prediction between short peptides and HLA-II alleles. Recently, MARIA [16] and NetMHCIIpan [17] stand out as widely acknowledged tools available online, both designed to predict the binding affinity (BA, denoted as half-maximum inhibitory concentration >100 nM). However, relying solely on BA characteristics proves inadequate for describing the antigenic presentation profiling of the genuinely presented ligands. Thus, MARIA also incorporates multiple relevant features, including the gene expression levels and proteasome cleavage scores, to enhance the predictive performance [18], yielding relatively accurate and robust results. By leveraging MS datasets, appropriate deep learning algorithms can directly learn the information on exogenously processed and presented peptides from cells, achieving significant improvements in BA prediction [19, 20]. However, it is important to note that strong binding affinities do not always consistently signify that antigens are presented by HLA alleles, owing to the high false discovery rates. To mitigate this issue, recent studies have opted to select eluted ligands (EL) from alleles as true candidates [21, 22]. In contrast to BA datasets, EL datasets provide explicit interactions of presentation for immunogenic epitopes, allowing further exploration of allele-specific immunogens pertinent to autoimmunity and antitumor immunity. Racle *et al.* [23] compiled an integrated EL dataset comprising 77 189 unique peptides from 23 distinct cell lines and tissue samples, offering high-quality data suitable for model training. Subsequently, they developed a deep learning method coupled with a motif deconvolution algorithm to integrate the features including pan-allele and specific-allele peptide N- and C- terminal motifs, along with binding core offset preferences [24]. This approach provides more accurate and interpretable predictions for immunogenic epitope presentation. Nevertheless, EL prediction based solely on peptide-HLA interactions falls short in assessing immunogenicity, as only a fraction of the myriad candidates can elicit responses from T cells. To address this challenge, recent tools have emerged. Dhanda *et al.* introduced CD4episcore [25], an IEDB online tool, for predicting the allele-independent exogenous T cell immunogenicity (IM) at the population level. Furthermore, DeepNeo is a CNN-based method to learn the distal amino acid (AA) features of pMHC–TCR interactions directly from immunogenicity data [26]. TLimmuno2 [27], a transfer learning-based long short-term memory framework, combines the BA and IM information to enhance the predictive performance of the HLA-II immunogenic epitopes. Despite these advancements, existing predictors still encounter limitations in accurately inferring HLA-II bound epitopes that trigger T cell responses, primarily due to inappropriate learning algorithms and inadequate training data. Beyond considering experimental accuracy, most majority of predictive tools cannot effectively model the complex properties of the natural neoantigen presentation process. Thus, these models are confused about interpreting the immunogenicity mechanism of intrinsic immune system.

In this study, we introduce HLAIImaster, an attention-based deep learning frame with adaptive domain knowledge learning designed to accurately predict the immunogenicity of epitopes that bind to the HLA-II alleles. HLAIImaster’s epitope predictions are not only solely based on the in-depth mass spectrometry elution data but also consider the immunogenicity of the peptide, a key factor in provoking T cell responses. The proposed method includes a motif deconvolution algorithm to explore the binding core offset preference and incorporates several crucial biological variants, such as gene expression level and proteasome cleavability, for high-quality and reliable training data. We also apply a conditional domain adversarial network (CDAN) to transfer learned knowledge of presented epitopes from the antigenic domain to the immunogenic domain, enhancing cross-domain generalization. HLAIImaster has been applied to diverse human cancer neoantigens, and the robust and accurate results align with previous studies, indicating that the predicted neoantigens are most likely to induce a corresponding immunogenicity reaction upon vaccination. Overall, the development of HLAIImaster addresses the long-standing challenge of identifying the immunogenic HLA-II antigens, offering significant clinical benefits for various cancers and autoimmune diseases.

## Results

### Development of HLAIImaster

**Figure 1:**
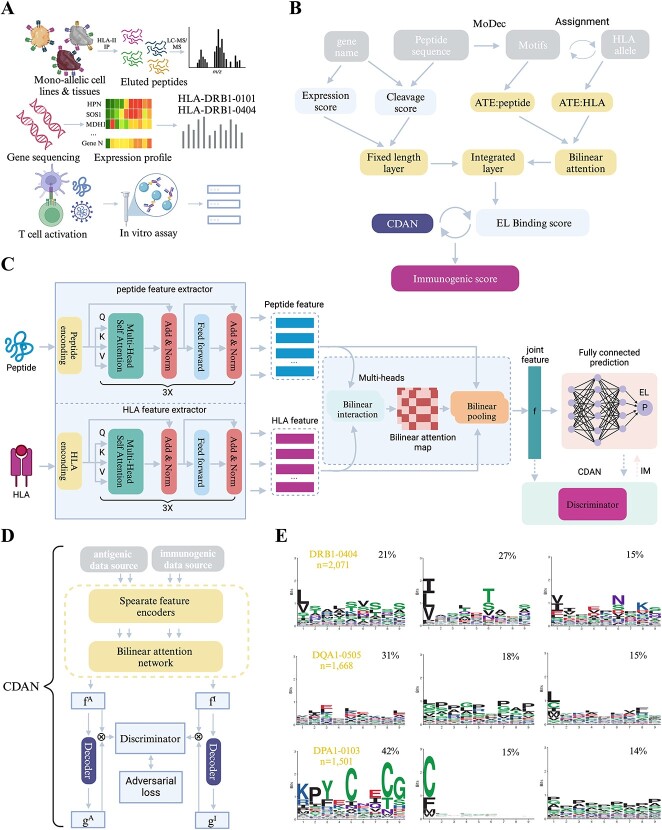
**Rationale and framework for the HLAIImaster**. (**A**) collection of various types of HLA II ligands used for training data; (**B**) workflow of HLAIImaster for predicting HLA-II epitope immunogenicity; (**C**) the HLAIImaster deep neural network architecture, and this sequence-only model takes in a pair of short peptide sequence and HLA sequence; (**D**) structure of the CDAN applied to transfer knowledge from the antigenic domain into the immunogenic domain; (**E**) motif deconvolution of motifs and their distribution across different HLA alleles.

Our objective was to enhance existing HLA-II prediction models by developing HLAIImaster using datasets from MS-based eluted ligand presentation profiling (Fig. 1A). HLAIImaster is a two-stage predictive method designed to determine the immunogenicity of HLA-II epitopes (Fig. 1B–D, See online supplementary material for a colour version of this Supplementary Fig. 1 and Methods). Firstly, we trained a peptide-HLA class II EL model employing augmented transformer encoders and a bilinear attention network to extract the feature representations of the peptide-HLA pairs [28, 29]. Subsequently, the EL model was transferred, and we utilized a CDAN to learn the immunogenic domain knowledge of the presented epitopes based on immunogenicity data [30]. The IM model was classified by consecutive fully connected layers to determine the HLA-II immunogenicity of epitopes. Additionally, we introduced some other pivotal variables related to the antigen presentation process, such as gene expression levels, protease cleavage scores, and binding core offsets [14]. Empirically, we defined the incremental contribution weights for corresponding predictor variables, i.e. peptide sequence (60%), gene expression (12%), cleavability (12%), and binding core offset (16%). The binding core offset, with length of nine AAs, was obtained using MoDec [23], a motif deconvolution algorithm that also records the position weight matrices of peptides (Fig. 1E). Given the challenges linked with the high polymorphism of HLA-II EL’s length, we explored the motifs of all peptides and assigned them to each allele, effectively resolving the bias associated with variable-length sequence data [31].

**Figure 2:**
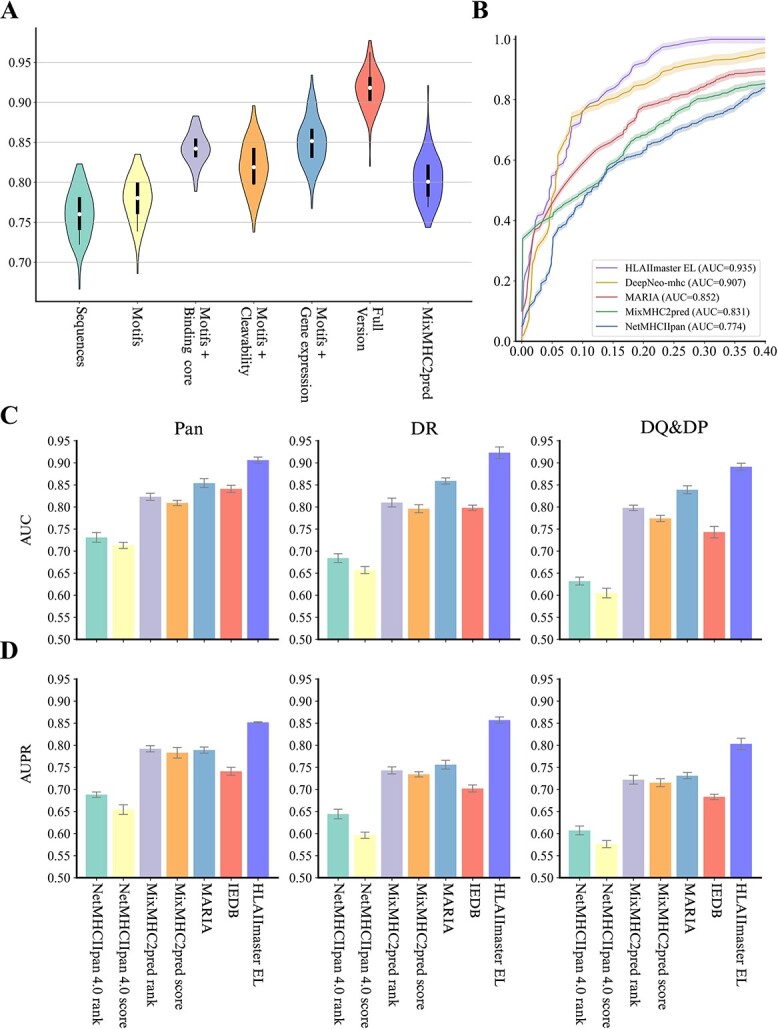
**HLAIImaster significantly improves class II epitope prediction**. (**A**) comparison of predictive performance of HLAIImaster, multiple biological variables, and MixMHC2pred; (**B**) performance of HLAIImaster and four alternative tools for K562 DBR1:01:01 ligand prediction; (**C** and **D**) comparison results of pan-allele, DQ-specific, and DQ&DP-specific models with existing HLA-II epitope predictive tools.

Initially, we utilized the same EL data used to train NetMHCIIpan-4.0 to construct the HLAIImaster EL model rather than relying solely on *in vitro* HLA binding affinities, which were insufficient for inferring the utility of antigen presentation. These EL data covered 72 class II alleles and comprised 223 931 measurements of peptide-HLA pairs. We then employed the immunogenicity data downloaded from the IEDB [32] and MHCBN [33] to train the HLAIImaster IM model on learning the immunogenicity of epitopes (See online supplementary material for a colour version of this Supplementary Table 1). The statistical information of immunogenicity data is presented in Supplementary Fig. 1a (See online supplementary material for a colour version of this figure). The input of the proposed model includes the peptide sequence, HLA sequence, gene expression level, and protease cleavage score. The final output is a probability score to infer whether the epitope is presented by the HLA molecule (EL model) or can provoke a CD4+ T cell immune response (IM model). We employed 10-repeated 5-fold cross-validation to train the proposed model and filtered out any tested peptides that occurred in the training or validation sequences. Additionally, apart from evaluating the prediction accuracy of the full version of HLAIImaster, we also investigated other models that trained on each combination of biochemical variants [34]. According to the results, our predictor showed significant improvement when considering the multiple features, with an average of 0.923 for HLAIImaster EL versus 0.805 for MixMHC2pred (Fig. 2A).

### Epitope presentation and immunogenicity prediction

To survey whether HLAIImaster was appropriate for HLA class II epitopes, we initially constructed the EL model using a HLA-DR dataset ($n=2071$) experimentally identified via MS from a human cell line (K562) expressing a single HLA-DR allele DRB1:04:04 [35]. This dataset mirrors the one used to test MARIA’s ability to predict the peptides presented by human APCs. In this evaluation, HLAIImaster EL was compared with four state-of-the-art predictive tools, i.e. DeepNeo-mhc [27], MARIA [16], MixMHC2pred [24], and NetMHCIIpan-4.0 [17] (Supplementary Note 1). The results, depicted in Fig. 2B, revealed that the improvement of HLAIImaster is particularly compelling in EL prediction, achieving a 0.935 value of Area Under the Receiver Operating Characteristic curve (ROC-AUC, “Materials and Methods”, Supplementary Note 2), which is 0.028 higher than the runner-up method, DeepNeo-mhc. Although other models also exhibited relatively effective performance in predicting the HLA-II bound peptides, they failed to elucidate the underlying biological characteristics due to the shallow structural design of neural networks.

The distributions of HLA-DR, DP, and DQ ligands with different lengths play central roles in the presentation process. Thus, we examined the peptide-extrinsic properties by categorizing the HLA loci into three models, namely the pan-allele model, the DR-specific model, and the DQ&DP-specific model. Of note, we included percentile rank [36] for existing tools and employed the IEDB predictor for better comparison. The effectiveness of three HLA loci models for each predictor was displayed (Fig. 2C,D). HLAIImaster EL demonstrated robust performance across different HLA loci models, whereas the performance of other predictors declined sharply in the DR-specific model, particularly in the DQ&DP-specific model. The balanced accuracy (B.Acc) in the DQ&DP-specific model for each tool was as follows: HLAIImaster (0.818), NetMHCpan-4.0 ranks (0.618) and scores (0.572), MixMHC2pred ranks (0.716) and scores (0.694), MARIA (0.718), and IEDB (0.664) (“Materials and Methods”, Supplementary Fig. 4a,b). We further investigated how the high variability in the length of HLA-II peptide ligands influenced the predictive performance (Supplementary Fig. 4c). After applying this more elaborate stratification (12-19 AA), HLAIImaster yielded the highest mean AUC and Area Under the Precision-Recall curve (AUPR) metrics of 0.871 and 0.749, respectively. In comparison, MixMHC2pred obtained the mean AUC and AUPR of 0.773 and 0.626, as well as MARIA [16] obtained the mean AUC and AUPR of 0.789 and 0.639, respectively. Although these two predictors showed relatively competitive performance due to considering multiple variants to learn the latent features of HLA II epitopes, HLAIImaster EL still demonstrated superiority. According to the results, HLAIImaster is most effective when the peptide length is 15, which is the optimal length for the HLA-II molecule presentation. Despite a significant decline in predictive performance as the peptide length grows, HLAIImaster still outperforms the compared predictors across different peptide lengths. In summary, HLAIImaster EL surpasses these state-of-the-art predictive tools across all metrics in both HLA loci and peptide length stratifications. Two-tailed paired $t$-tests applied to the epitope presentation confirm that the improvements of the HLAIImaster EL model are statistically significant ($P<$.001).

**Figure 3:**
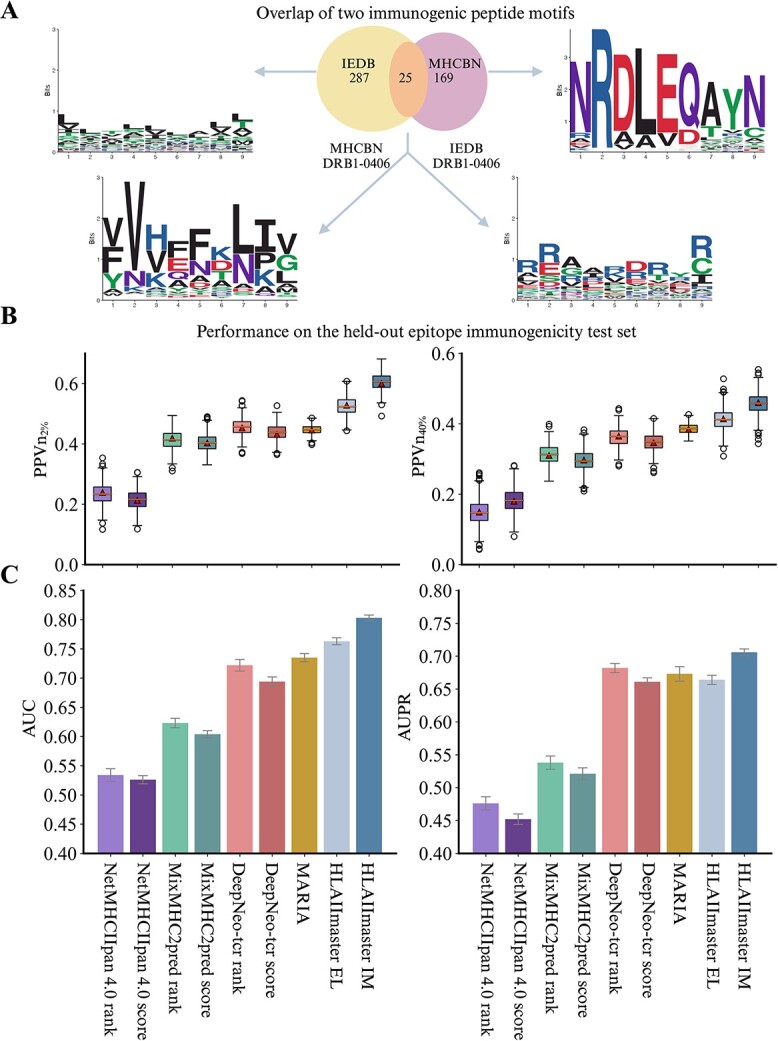
**HLAIImaster can accurately identify the class II epitope immunogenicity**. (**A**) overlap and sequence motifs of two immunogenic epitope sets; partial peptides ($n=25$) were shared in both IEDB($n=287$) and MHCBN ($n=169$) sets; (**B**) box plots of $PPVn_{2\%}$ and $PPVn_{40\%}$ are calculated for each model as the fraction of neoepitopes that are immunogenic within top $n$ predictions; (**C**) AUC and AUPR hist plots for HLAIImaster and existing predictors on the held-out epitope immunogenicity test set.

Next, we evaluated the prediction of immunogenicity on a short list of candidate neoepitopes capable of eliciting clinical immune responses [32, 33]. The immunogenicity dataset comprises 7853 candidate samples (4384 immunogenic and 3469 non-immunogenic), which were experimentally verified through multiple human *in vivo* assays for immunogenic reactivity. To balance the positive-to-negative ratio in the IM dataset, we randomly generated 14 067 length-matched decoys from the human proteome [37] as negative examples, as the number of non-immunogenic peptides often far exceeds immunogenic ones in practice. Interestingly, the full potential motifs and overlaps ($n=25$) across the two datasets (IEDB and MHCBN) were identified from the immunogenic neoepitopes (Fig. 3A). Details regarding the data cleaning process can be found in Materials and Methods section. Subsequently, we used a held-out test to assess the predictive capability of the HLAIImaster IM model for the immunogenicity of neoepitopes (Fig. 3B,C).

In this evaluation, we utilized the positive predictive value of the top $n$ correct candidate neoepitopes, referred to as PPVn. This is a widely accepted and persuasive metric for measuring the prediction precision [13, 15]. To calculate PPVn, we considered the fraction of actual immunogenic neoepitopes that scored within the top 2% and 40% (“Materials and Methods”). The results of this precision calculation are displayed in Fig. 3B; we draw the box plot for $PPVn_{2\%}$ and $PPVn_{40\%}$ with all top $n$ candidates that a perfectly predictive tool can truly identify. In terms of $PPVn_{2\%}$ metrics, HLAIImaster achieved a value of 0.607, which was surprisingly superior to the best previous predictor; DeepNeo-tcr rank, which achieved a value of 0.457. Furthermore, the results indicated that the well-designed CDAN for adoptive domain knowledge significantly outperformed the HLAIImaster EL model, which obtained the $PPVn_{2\%}$ value of 0.524. However, the $PPVn_{40\%}$ performance of all the models decreased sharply, which aligns with the common sense that precision tends to be compromised as the fraction of top $n$ hits increases. Specifically, HLAIImaster yielded this precision of 0.457 and 0.412 on IM and EL models, respectively, whereas the second-best method MARIA achieved 0.387 and the third one (DeepNeo-tcr) achieved 0.363 in terms of $PPVn_{2\%}$ ($P<$.05). We hypothesized that HLAIImaster IM is more likely to struggle with HLA-DP epitope identification due to insufficient examples for adaptive domain knowledge learning.

In addition to precision, we recorded four metrics, i.e. AUC, AUPR, F$_{1}$-score, and B.Acc (“Materials and Methods”) to systematically compare the immunogenic identification performance of HLAIImaster with alternative methods. The AUC and AUPR values for the immunogenicity of neoepitopes were illustrated in Fig. 3C, while the F$_{1}$-score and B.ACC values were in Supplementary Fig. 5a,b. Immunogenic neoepitope prediction ablated by epitope length was also presented (Supplementary Fig. 5c). HLAIImaster IM achieved the highest AUC score at 0.802, with MARIA trailing behind by a 0.07 performance drop. Meanwhile, HLAIImaster EL secured the second-best position in inferring whether a neoepitope has immunogenicity to mediate T cell recognition, with an AUC score of 0.763. Additionally, HLAIImaster IM significantly outperformed all previous methods in terms of AUPR, achieving a value of 0.706, whereas the next best method, MARIA, achieved an AUPR of 0.673. Intuitively, the IM model yielded F$_{1}$-score and B.Acc values of 0.621 and 0.752, respectively, while the EL model yielded values of 0.587 and 0.683, further indicating a significant enhancement after domain knowledge transfer learning. Robust domain knowledge learning that exhibits a clinical context differentiation of immunogenic neoepitopes will contribute to the clinical setting and medical peculiarities of cancers, enabling future neoantigen-based therapies to provide greater clinical benefit. This immunogenicity evaluation was conducted using two-tailed paired $t$-tests ($P<$.001, statistically significant).

### HLA attention visualization

One of the additional advantages of HLAIImaster is to empower the molecular-level insights and interpretable significance for immune response efforts. This is achieved by employing bilinear attention networks to visualize the contribution of each AA residue of HLA for final classification. Specifically, we represented the relay node attention as a heatmap overlay on the crystal structure of the HLA loci of interest. The attention for each AA residue per HLA allele in the EL dataset is described in Supplementary Fig. 6a. Furthermore, we generated 3D structure models of HLA proteins with attention coloring by using PyMol [38] and AlphaFold [39] tools to highlight the HLA locus that achieved the best AUPR performance (Supplementary Fig. 6b). By visualizing the HLA encoding pesudosequences, we can observe the AAs in the antigenic binding groove, which are crucial elements for interacting with peptides [40]. Therefore, the general binding core preference of class II HLA molecules can be imputed for a better understanding of the presentation of immunogenic neoepitopes. For example, the HLA allele DRB1:07:01 is more likely to recognize and bind with breast cancer-specific neoantigens with high affinities, consistent with previous studies [41, 42]. These findings demonstrate that HLAIImaster is capable of identifying important AA positions and offering an interpretable mechanism for epitopes on the HLA surface.

### HLAIImaster identifies diverse cancer neoantigens

**Figure 4:**
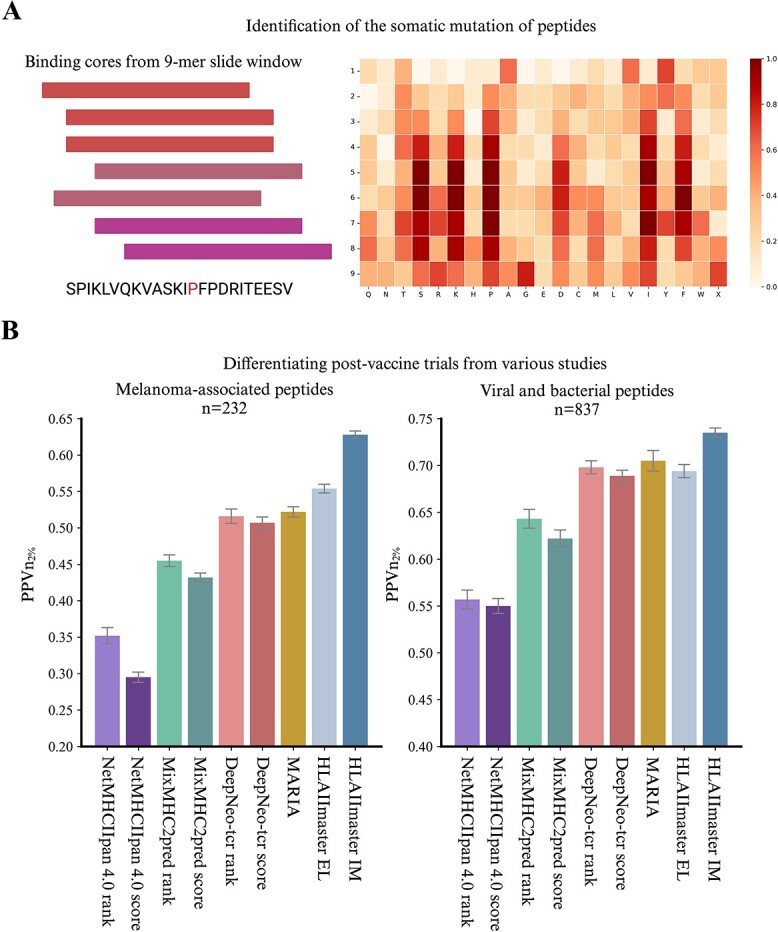
**HLAIImaster identifies diverse cancer neoantigens in real-world applications**. (**A**) identification of somatic mutations in neoantigens through the 9-mer binding core sliding; (**B**) performance of each model in differentiating post-vaccine CD4+ T cell responses from various studies, left: melanoma-associated peptides ($n=232$); right: viral and bacterial peptides ($n=837$).

Finally, we investigated the capability of HLAIImaster to identify immunogenic neoantigens, viral, and bacterial proteins (Supplementary Table 2). We focused on personalized protein-coding somatic mutations, such as non-synonymous single-nucleotide variants, nucleotide insertions or deletions (indels), and gene fusions, which are more attractive vaccine candidates for cancers [43], particularly melanoma. HLAIImaster was applied to analyze two datasets of personalized melanoma vaccine neoantigens ($n=232$ in total) with experimentally wet-lab validation (*in vitro* CD4+ T lymphocyte enzyme-linked immunospot) [44, 45]. Our analysis revealed that the gene expression levels of immunogenic neoantigens and non-immunogenic neoantigens candidates were mostly indistinguishable, which is consistent with MARIA. To determine the actual binding core of epitopes, we utilized HLAIImaster on the 18-amino-acid oligomer with a 9-mer slide window (Supplementary Fig. 7). The results indicated that HLAIImaster could accurately predict the actual binding core (Fig. 4A).

Top hits from HLAIImaster and from other advanced predictors were also evaluated for HLA-II immunogenicity (Fig. 4B). The results showed a higher fraction $PPVn_{2\%}$ among the predictions of HLAIImaster EL versus MARIA (0.554 for HLAIImaster EL versus 0.522 for MARIA). The HLAIImaster IM model yielded the highest $PPVn_{2\%}$ value of 0.628, which is significantly improved against HLAIImaster EL. Transfer learning through the CDAN framework effectively captured the immunogenic features of melanoma-associated CD4+ T cell neoepitopes. For the viral and bacterial peptides [4, 41, 46, 47], we also tested them with CD4+ T cells from two melanoma patients ($n=837$, “Materials and Methods”). Our proposed method outperformed other predictive tools in both EL and IM models. Specifically, HLAIImaster IM and EL models achieved the $PPVn_{2\%}$ value of 0.734 and 0.695, almost acquiring 1.3-fold improvement compared with canonical model NetMHCIIpan-4.0. These results highlighted the promising potential of HLAIImaster in prioritizing class II HLA neoepitopes most likely to elicit tumor-specific CD4+ T cell immune reactivity.

### Discussion

Empirically, predictors for class II HLA binding have largely relied on *in vitro* peptide-binding assay datasets for training. However, when considering HLA-II binding affinities alone, predicted results are not sufficient to accurately prove whether the presented antigens can elicit downstream immune responses. By combining large-scale eluted HLA-II peptidomics with a motif deconvolution algorithm, we developed an optimized framework based on unbiased MS profiling of HLA-II ligands for exogenous epitope predictions. To accurately quantify the immunogenicity of HLA-II ligands, we incorporated the experimentally verified immunogenic training data from the IEDB and MHCBN. HLAIImaster, an attention-based deep learning framework with adaptive domain knowledge, is designed to integrate heterogeneous features and variable-length sequences for class II epitope presentation prediction and adopt transfer learning techniques, i.e. CDAN, for high-confidence immunogenicity prediction. Experimental results demonstrate that HLAIImaster is superior to existing models in most validation cohorts. The significant improvement of HLAIImaster models can be attributed to four main factors. (i) The proposed models are trained on both in-depth MS-eluted ligand data and *in vitro* verified immunogenicity data from monoallelic cell lines. (ii) MoDec is utilized to explore the epitope’s binding core offset preference in the natural presentation process. (iii) Several critical biological features, such as gene expression levels and protease cleavage scores, are integrated into the model. (iv) Adaptive domain knowledge learning enhances cross-domain generalization of epitope immunogenic responses, enabling future neoantigen-based therapies to provide greater clinical benefit. Moreover, allele-specific and pan-allele models are stratified to address the complex situations of specific or uncharacterized alleles.

Furthermore, the HLA-II gene expression analysis provided a more comprehensive understanding of APCs or tumor HLA-II presentation in pan-cancer (Supplementary Fig. 2). According to the HLA-II cleavage signatures, we observed the enrichment of ligand flanking sequences for proline or alanine within two residues, consistent with that proline enrichment influences peptide processing and loading [16, 24, 48] (Supplementary Fig. 3). Experimental results also demonstrate how HLAIImaster might enable immunologists and biologists to better infer immunogens related to antitumor immunity or autoimmunity. Due to the inherent challenges still limiting the accuracy of existing tools for predicting tumor-specific HLA-II ligands, HLAIImaster should grant scientists the ability to learn the non-canonical HLA-II neoantigens (more likely to induce malignancies than canonical ones) [43, 49]. This means that if we want HLAIImaster to achieve near-perfect accuracy for prioritizing cancer vaccine candidates, the NGS techniques (e.g. Ribo-seq, RNA-seq, and scTCR-seq) of tumors should be combined to enhance the qualitative and quantitative training data [50]. Thus, by integrating in-depth high-throughput identified T cell reactive data and deep learning, we believe that a more comprehensive and accurate method will be developed for the recognition of neoantigens, which play central roles in antitumor immunity and personalized cancer vaccines. Finally, HLAIImaster implementation and data sources are freely available on the GitHub website https://github.com/TomasYang001/HLAIImaster, and we believe that the introduced method can contribute to the community for tumor immunogenomics research.

## Materials and methods

### Datasets

#### Epitope presentation data

The presentation training data spanned 72 class II alleles and comprised 223 931 MS-derived ELs and 1 640 832 decoys. The training dataset was built upon the NetMHCIIpan-4.0 monoallelic EL datasets. We split these instances into a training set (hits = 179 144; decoys = 1 312 665), and a validation set (hits = 44 787; decoys = 328 167). The presentation evaluation set comprised 50 382 ELs and 544 395 decoys. These are the same monoallelic EL samples that were used in the NetMHCIIpan-4.0 test dataset but unobserved in the training data.

#### Immunogenicity data preparation

We collected human immunogenicity data from IEDB and MHCBN on or before 15 May 2022. More specifically, we used the keywords: linear peptide, T cell, MHC-II, human and any disease, and selected data that measured T cell activation through Interferon-$\gamma $ secretion. We then employed high-quality data processing, (i) data samples without explicit four-digit HLA types were removed, (ii) the peptide length was restricted to the range of 11-19 mer, (iii) data that have no clear experimental information to support the immunogenic labels were discarded, and (iv) data with contradicting experimental results were treated as positive candidates considering the fluctuant nature of the epitope immunogenicity experiment. Since HLA-DP and HLA-DQ are heterodimers, experimental data were thus given as HLA-DQA/HLA-DQB pairs. The HLA-DPB allele was removed from the model because of the inadequacy of data. Eventually, we obtained 4384 positive data points and 3469 negative data points for HLA II.

### Integration of HLA-II peptidomics

#### Motif deconvolution algorithm

The HLA-II ligands are of different lengths, are coming from different alleles, and are more promiscuous than HLA-I, and their core positions are prior unknown in the binding groove, which could make identifying binding motifs more challenging. To address this complexity, we employed a probabilistic motif deconvolution algorithm (MoDec), as previously developed, which allows predictors to learn various motifs anywhere in the HLA-II peptidomics, including the position weights and binding core offset preferences. Here, we set the motif length as 9, considering that HLA-II is more likely to bind ligands with a nine-amino-acid core. Additionally, we extended the peptides by incorproating three AAs upstream of the N terminus and downstream of the C terminus to capture the properties of naturally presented HLA-II ligands (done with MoDec). For the ligand motifs from IEDB, only the peptides labeled as “positive-high” binders were retained. In this study, ligand motifs were defined through MoDec using default sets (250 runs).

#### Gene expression analysis

In general, when personalized RNA-seq expression profiles were not available, gene expression values were estimated from the median of TCGA RNA-seq results of the closest cancer type. We normalized these expression values into transcripts per million (TPM, fixed at 50) to facilitate explicit comparisons across studies. Each gene TMP value was transformed into logarithmic space using the formula $log10(X+ 10^{6})$. Noting that peptide sequences without known RNA-seq gene expression values were excluded from the analysis. The analysis of this gene expression profile was conducted following the method proposed by Chen *et al.* [16].

#### Cleavage score estimation

Proteolytic cleavability preferences play a crucial role in the antigen presentation process. To account for this, we compared the frequency of AA around class II peptide cleavage sites with a background distribution. The cleavage sites contained three AA upstream and three AA downstream of the HLA-II peptide N and C termini, arranged in an N to C terminus order. We generated an equal number of length- and gene-matched decoys based on our HLA-II epitope data and used the AA frequency of the identical cleavage sites as the background distribution. We then constructed a fixed neural network to quantitatively measure the protease cleavage signatures of flanking residues of gene-peptide pairs. This model encodes these 6 AA sequences (three AAs upstream and three AAs downstream of the query peptide N and C terminus selected in a human proteome dictionary) with two fully connected layers of 32 units and outputs a cleavage score ranging from 0 to 1 (Supplementary Fig. 1d).

### Predictive performance metric calculation

To evaluate the predictive power of our model, we employed several key metrics. For the EL model evaluation, the AUC score, AUPR score, F1-score, and B.Acc were used. We advocated for the PPVn metric of Abelin *et al.* instead of the commonly used one to evaluate the IM model. Because it is more suitable for the HLA-II epitope prediction problem space where a small number of hits need to be recognized among a huge number of nonbinders. In our IM data (n), each binder was accompanied by 98 random decoys from the human proteome. We then considered the fraction of correctly predicted binders in the top 2% of the dataset ($PPVn_{2\%}$), the same for ($PPVn_{40\%}$). For a detailed introduction and mathematical formulation of these evaluation criteria, please refer to the supplementary materials.

### Allele and peptide sequence encoding

Three main amino acid sequence encodings were utilized to represent the peptide and allele pseudosequence: (i) traditional one-hot encoding; (ii) preferential interaction encoding based on the $C\alpha -C\alpha $ distance; and (iii) similarity encoding using the BLOSUM62 substitution matrix. Specifically, the one-hot encoding represents a peptide as a (L, 21) 2D matrix (where $L$ is the length of the peptide), with each amino acid in this peptide is a 21-numerical vector (20 amino acids $+$ padding X). The $C\alpha -C\alpha $ distance reflects the physical interactioin between each amino acid distal surface in space. The blosum62 matrix provides a common definition for amino acid pseudosequence representation. Noting that, for allele representation, we used the 269 amino acid residues for HLA in preferential interaction encoding and only 34 amino acid residues are included in one-hot or similarity encoding. Attempts were made to employ PMBEC [51] and ProtVec [52] sequence encodings, but their influence on the predictive performance was inconspicuous.

### HLAIImaster model

The majority of existing HLA-II epitope predictors solely build upon the binding information of recombinant HLA-II alleles to peptides, lacking immunogenic features. In contrast, HLAIImaster aims to explicitly model the knowledge of the antigenic domain of ELs presented by HLA-II alleles and transfer it into the immunogenic domain using CDAN. Rather than *in vitro* binding assays, MS-derived EL data from APCs were utilized as the true measurements. For the convenience of comparison with other predictors, we also converted the binding score into a percentile rank per peptide.

HLAIImaster is a two-stage attention-based deep learning prediction model, namely HLAIImaster EL and HLAIImaster IM. In our framework, peptide sequences (longer than 12 AAs and shorter than 19 AAs), cell or patient HLA alleles, and gene names are taken as input to identify the epitope presentation. MoDec first deconvolves the motifs (the key for natural presentation in the HLA binding groove) of all peptides to obtain the binding core offset preference and protein weights. HLAIImaster estimates cleavage scores using a separate neural network, as mentioned above. Gene expression signatures of each gene are also estimated using a public profile dictionary of gene expression on the basis of the tissue-matched RNA-seq results from TCGA (Supplementary Fig. 2c).

The HLAIImaster EL model is trained on an epitope presentation dataset, splitting into a 4:1 ratio for the training set and a validation set. Artificial negative peptides (decoys) are generated from the human proteome, ensuring no overlap with the positive samples (hits). Training follows a standard 10-repeated five-fold cross-validation approach. To be specific, HLAIImaster EL contains two separate transformer encoder blocks to extract the biological characteristics of HLA pseudosequences and peptide sequences (Supplementary Fig. 1c). Three consecutive augmented transformer encoders are used in the HLA or peptide feature extractor with a multi-head attention mechanism. The number of heads is set to 12 after careful selection to enrich the underlying features. Subsequently, a bilinear attention network module is used to learn their joint representation. The bilinear attention network captures pairwise local interactions between alleles and peptides, utilizing a bilinear pooling layer to jointly represent the relationships of HLA peptide pairs. Afterward, the learned representations are integrated with three fully connected dense layers (128, 64, 32 units). The output layer comprises two units indicating presenting (1 or True) and non-representing (0 or False). HLAIImaster EL optimizes itself using the AdamW strategy to minimize binary cross-entropy loss. The optimizer’s learning rate is adjusted from the initial setting of $10^{-5}$ to $10^{-2}$ by maximizing model performance on the validation set, and other AdamW hyperparameters are set by default. To maximize the predictive effectiveness, all hyperparameters are carefully selected based on rigorous grid research.

Deep learning models tend to perform well on similar data from the same distribution (e.g. in-domain) [16, 23, 53] but poorer on dissimilar data with different distribution (e.g. cross-domain). It is a key challenge to improve model performance on cross-domain epitope immunogenicity prediction. More importantly, intricate process of antigen presentation and recognition in immune system may lead to exaggerated distribution shifts. However, conditional domain adversarial networks have been proven to be effective in enhancing the performance of cross-domain data distribution. Thus, we explored HLAIImaster IM with a CDAN module based on transfer learning, which is the CDAN module that contains three main components: (i) the antigenic epitope feature extractor $F(\cdot )$; (ii) the immunogenic decoder $G(\cdot )$; and (iii) the adoptive domain discriminator $D(\cdot )$. $F(\cdot )$ was used to indicate the separate feature encoders and bilinear attention network together to generate joint representations of input domain data, which is $f^{A}$ and $f^{I}$. $G(\cdot )$ was constructed with two fully connected layers and classified by a softmax function to obtain a classifier prediction $g^{A}$ and $g^{f}$. Furthermore, a multilinear map was utilized to capture multiplicative interaction $h$ between two independent data distributions (antigenic domain knowledge from $f$ versus immunogenic domain knowledge from $g$).

The adoptive domain discriminator $D(\cdot )$, consisting of a three-layer fully connected networks, learns to differentiate whether a joint conditional representation $h$ is from the antigenic domain or the immunogenic domain. Inversely, the epitope feature extractor $F(\cdot )$ and the immunogenic decoder $G(\cdot )$ were trained to confuse the discriminator $D(\cdot )$ and minimize the antigenic domain cross-entropy loss $\mathcal{L}_{a}$ with EL label information. Also, we calculated the adversarial loss $\mathcal{L}_{adv}$ for domain discrimination. Thus, the optimization problem was formulated as a minimax paradigm 


(1) \begin{align*}& \mathop{min}\limits_{D} \mathop{max}\limits_{F,G} \mathcal{L}_{a}(F,G) - \omega \mathcal{L}_{adv}(F,G,D),\end{align*}

where $\omega> 0$ is a hyperparameter to weight $\mathcal{L}_{adv}$. By using adversarial training, the HLAIImaster IM effectively mitigates the data distribution of these two domains, thereby enhancing the generalization of neoepitope immunogenicity prediction. Training was performed for 32 batches and 30 epochs.

HLAIImaster was developed using Python 3.8.16 and Pytorch 1.12 on CUDA 10.2 with two NVIDIA RTX 3090 GPUs (24GB of RAM). For general applications, a 2 GHz Intel Core CPU is sufficient.

### Analyzing cancer neoantigen candidates with HLAIImaster

To further investigate whether HLAIImaster can assess the tumor-specific non-synonymous mutations in protein-coding genes, we retrieved a list of known tumor-associated, viral, and bacterial peptides in two cancer vaccine trials and one healthy donor (HLA-DRB1:07:01-positive). Each somatic mutation was represented in an 18-AA oligomer to include all possible 9-AA binding cores. In this analysis of therapeutic cancer vaccine candidates, the TPM was fixed at 50 to model the high density of mutant peptides resulting from vaccinations. The highest score among all binding cores was determined for each tested somatic mutation. Of note, neoantigen examples without supported HLA-II alleles were excluded from our analysis.

### Statistical analysis

HLA-II motifs and corresponding logo plots were generated with MoDec and ggseqlogo [54], respectively. Figures’ plotting in this work was created by matplotlib and seaborn package. All evaluation metrics were computed by the scikit-learn python package [55]. All statistical significances were calculated in two-sided tests and done in an R environment.

**Key Points**

- HLAIImater, an attention-based deep learning method with adoptive domain knowledge, is introduced to accurately identify the immunogenicity of HLA-II epitopes, which is crucial for CD4+ T cell immunogenic responses.
- Applying a motif deconvolution algorithm to explore the preferential binding core offsets in the natural presentation process, and integrating several important biological features, such as gene expression levels and proteasome cleavage scores, for comprehensively characterizing class II HLA neoepitopes.
- HLAIImaster exhibits significant improvement against existing predictive tools for epitope immunogenicity, regardless of the allele- or length-specific model.
- Neoantigens caused by tumor-specific non-synonymous mutations can be identified using HLAIImaster for antitumor immunity or personalized cancer vaccine.

## Supplementary Material

Supplementary_Material_bbae302(1)

Supplementary_Table_1_bbae302

Supplementary_Table_2_bbae302

## Data Availability

The large-scale eluted HLA-bound Ligandomes are available at https://services.healthtech.dtu.dk/services/NetMHCIIpan-4.0/. The immunogenicity epitope dataset is provided in Supplementary Table 1. The neoantigens across diverse studies are provided in Supplementary Table 2. TCGA RNA-seq results for gene expression analysis were obtained from the GDC Data Portal (https://portal.gdc.cancer.gov/). The implementation of HLAIImaster is publicly available at https://github.com/TomasYang001/HLAIImaster.

## Author Biographies

**Qiang Yang** is a master student at the School of Medicine and Health, Harbin Institute of Technology. His main research interests focus on deep learning algorithms and their applications in immunotherapy.

**Long Xu** is a PhD student at the School of Computer Science and Technology, Harbin Institute of Technology. His expertise is computational biology and machine learning.

**Weihe Dong** is a master student at the College of Computer and Control Engineering, Northeast Forestry University. His expertise is computational biology and machine learning.

**Xiaokun Li** is a PhD student at the School of Computer Science and Technology, Harbin Institute of Technology. His research interests include computational biology, artificial intelligence, cryptology, and information safety.

**Kuanquan Wang** is a professor at the School of Computer Science and Technology, Harbin Institute of Technology. His research interests are computational biology, image processing, and pattern recognition.

**Suyu Dong** is an associate professor at the College of Computer and Control Engineering, Northeast Forestry University. Her research interests are computational biology and machine learning.

**Xianyu Zhang** is a professor at the Department of Breast Surgery, Harbin Medical University Cancer Hospital. His research interests are monitoring and regulation of tumor genome evolution.

**Tiansong Yang** is a professor of the Department of Rehabilitation, The First Affiliated Hospital of Heilongjiang University of Traditional Chinese Medicine. Her research interests are immunotherapy and computational biology.

**Feng Jiang** is a professor at the School of Medicine and Health, Harbin Institute of Technology. His research interests are machine learning and biomedical engineering.

**Bin Zhang** is a post doctor at the Computer, Electrical and Mathematical Sciences and Engineering Division at the King Abdullah University of Science and Technology (KAUST), Saudi Arabia. His research interests are bioinformatics and computational biology.

**Gongning Luo** is a scientist at the Computer, Electrical and Mathematical Sciences and Engineering Division at the King Abdullah University of Science and Technology (KAUST), Saudi Arabia. His research interests are computational biology and machine learning.

**Xin Gao** is a professor of computer science in the Computer, Electrical and Mathematical Sciences and Engineering Division at the King Abdullah University of Science and Technology (KAUST), Saudi Arabia. His research interests are bioinformatics, computational biology, and machine learning.

**Guohua Wang** is a professor at the College of Computer and Control Engineering, Northeast Forestry University. His research interests are bioinformatics, machine learning, and algorithms.
