# Supplementary material for: HLAIImaster: a deep learning method with adaptive domain knowledge predicts HLA II neoepitope immunogenic responses
Source: Brief Bioinform. 2024 Jun 26;25(4):bbae302. doi: 10.1093/bib/bbae302 (PMC11200192; doi:10.1093/bib/bbae302)
Supplement: Supplementary_Material_bbae302(1) [file supplementary_material_bbae302(1).pdf]

OXFORD

Briefings in Bioinformatics, 2024
Supplementary Information
Problem Solving Protocol

PROBLEM SOLVING PROTOCOL

HLAIImaster: a deep learning method with adaptive domain knowledge predicts HLA II neoepitope immunogenic responses

Qiang Yang[1], Long Xu[2], Weihe Dong[3], Xiaokun Li[2, 4, 5, 6 *], Kuanquan Wang[2], Suyu Dong[3,*], Xianyu Zhang[7], Tiansong Yang[8], Feng Jiang[1], Bin Zhang[9], Gongning Luo[9], Xin Gao[9] and Guohua Wang[3,*]

[1]School of Medicine and Health, Harbin Institute of Technology, Yikuang Street, 150000, Harbin, China.
[2]School of Computer Science and Technology, Harbin Institute of Technology, West Dazhi Street, 150001, Harbin, China.
[3]College of Computer and Control Engineering, Northeast Forestry University, Hexing Road, 150004, Harbin, China.
[4]School of Computer Science and Technology, Heilongjiang University, Xuefu Road, Harbin 150080, China.
[5]Postdoctoral Program of Heilongjiang Hengxun Technology Co., Ltd., Xuefu Road, 150090, Harbin, China.
[6]Shandong Hengxun Technology Co., Ltd., Miaoling Road, 266100, Qingdao, China.
[7]Department of Breast Surgery, Harbin Medical University Cancer Hospital, Haping Road, Harbin 150081, China.
[8]Department of Rehabilitation, the First Affiliated Hospital of Heilongjiang University of Traditional Chinese Medicine, and Traditional Chinese Medicine Informatics Key Laboratory of Heilongjiang Province, Harbin 150040, China.
[9]Computer, Electrical and Mathematical Sciences & Engineering Division, King Abdullah University of Science and Technology, 4700 KAUST, Thuwal 23955, Saudi Arabia.
*Corresponding authors:
Xiaokun Li, School of Computer Science and Technology, Harbin Institute of Technology, 150001, Harbin, China; E-mail: lixiaokun@hlju.edu.cn; Suyu Dong, College of Computer and Control Engineering, Northeast Forestry University, 150004, Harbin, China; E-mail: dongsuyu@126.com; Guohua Wang, College of Computer and Control Engineering, Northeast Forestry University, 150004, Harbin, China; E-mail: ghwang@nefu.edu.cn

Supplementary Fig. 1

a

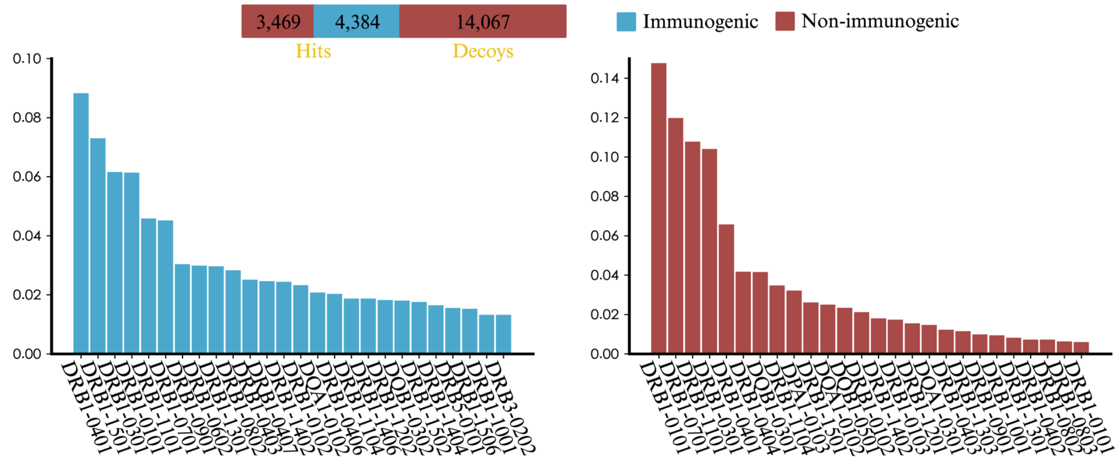

b

One-hot encoding for sequence EDSDKLFESKAELADHQKF

Animo Acid	P1	P2	P3	P4	P5	P6	P7	P8	P9	···	P19
Q	0	0	0	0	0	0	0	0	0	···	0
N	0	0	0	0	0	0	0	0	0	···	0
T	0	0	0	0	0	0	0	0	0	···	0
S	0	0	1	0	0	0	0	0	1	···	0
R	0	0	0	0	0	0	0	0	0	···	0
K	0	0	0	0	1	0	0	0	0	···	0
H	0	0	0	0	0	0	0	0	0	···	0
P	0	0	0	0	0	0	0	0	0	···	0
A	0	0	0	0	0	0	0	0	0	···	0
G	0	0	0	0	0	0	0	0	0	···	0
E	1	0	0	0	0	0	0	1	0	···	0
D	0	1	0	1	0	0	0	0	0	···	0
C	0	0	0	0	0	0	0	0	0	···	0
M	0	0	0	0	0	0	0	0	0	···	0
L	0	0	0	0	0	1	0	0	0	···	0
V	0	0	0	0	0	0	0	0	0	···	0
I	0	0	0	0	0	0	0	0	0	···	0
Y	0	0	0	0	0	0	0	0	0	···	0
F	0	0	0	0	0	0	1	0	0	···	1
W	0	0	0	0	0	0	0	0	0	···	0
X	0	0	0	0	0	0	0	0	0	···	0

c

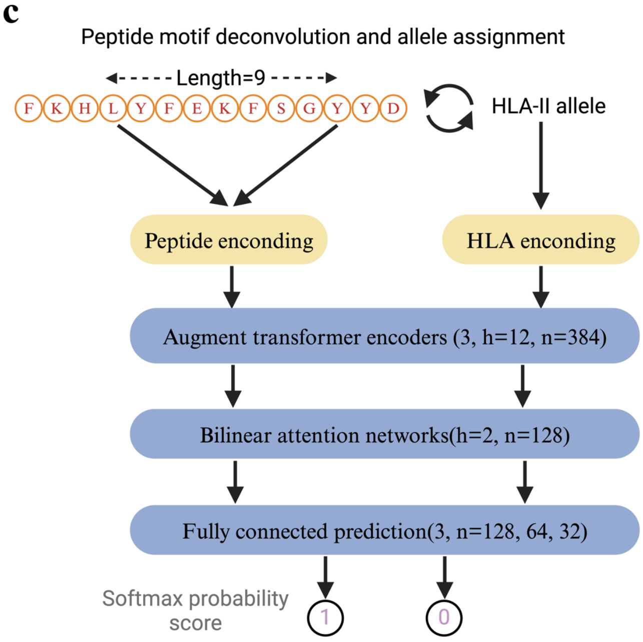

d

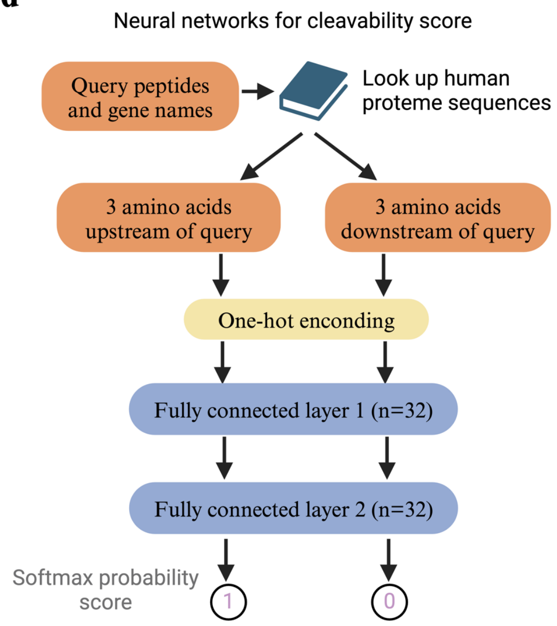

Supplementary Figure 1

Immunogenicity data processing and detailed individual neural network architectures.

(a) Summary of the immunogenicity data used to train the HLAIImaster. (b) An example (EDSDKLFESK AELADHQKF) of how variable length amino acid sequences (11-19AA) to be one-hot encoded for deep learning purposes. (c) Peptide motif deconvolution and allele assignment with a 9-mer slide window. The 9-mer peptide fragment that yields the best output is set to be the binding core. (d) The model architecture of peptide sequence cleavage scores for epitope presentation. The algorithm takes in a pair of query gene and short peptide sequence, and look up human proteome sequence database to determine the upstream and downstream three amino acid sequences (flanking sequences).

Supplementary Fig. 2

a

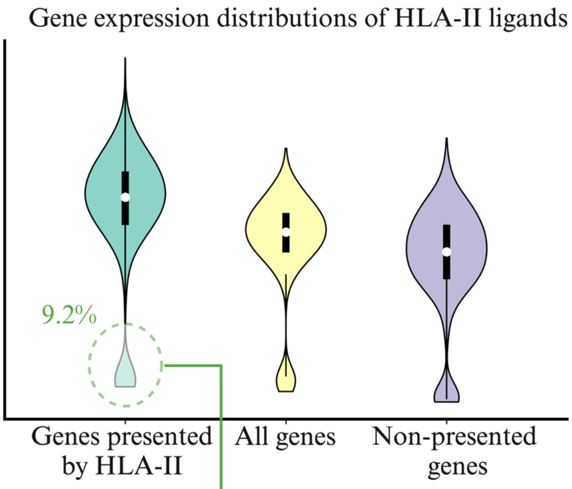

Cellular component enrichment	GO term ID	P value	Bonferroni's Q value
cell adhesion	GO:0007155	4.67E-09	2.17E-05
platelet degranulation	GO:0002576	6.04E-11	2.81E-07
enzyme inhibitor activity	GO:0004857	2.18E-05	2.12E-02
heparin binding	GO:0008201	4.22E-05	4.10E-02

b

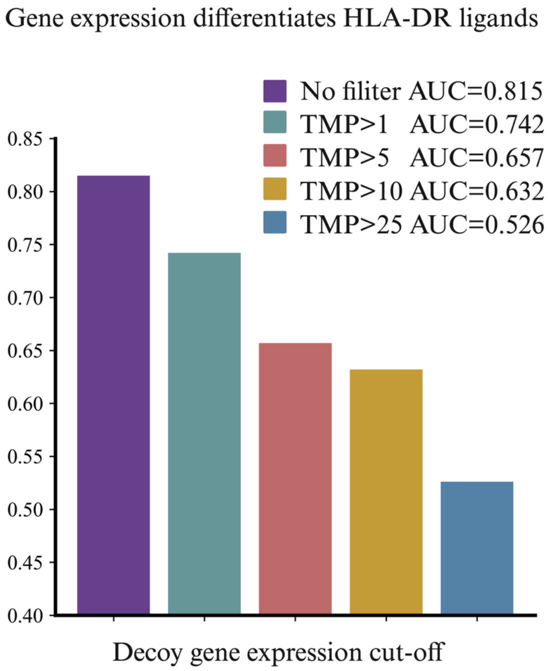

c

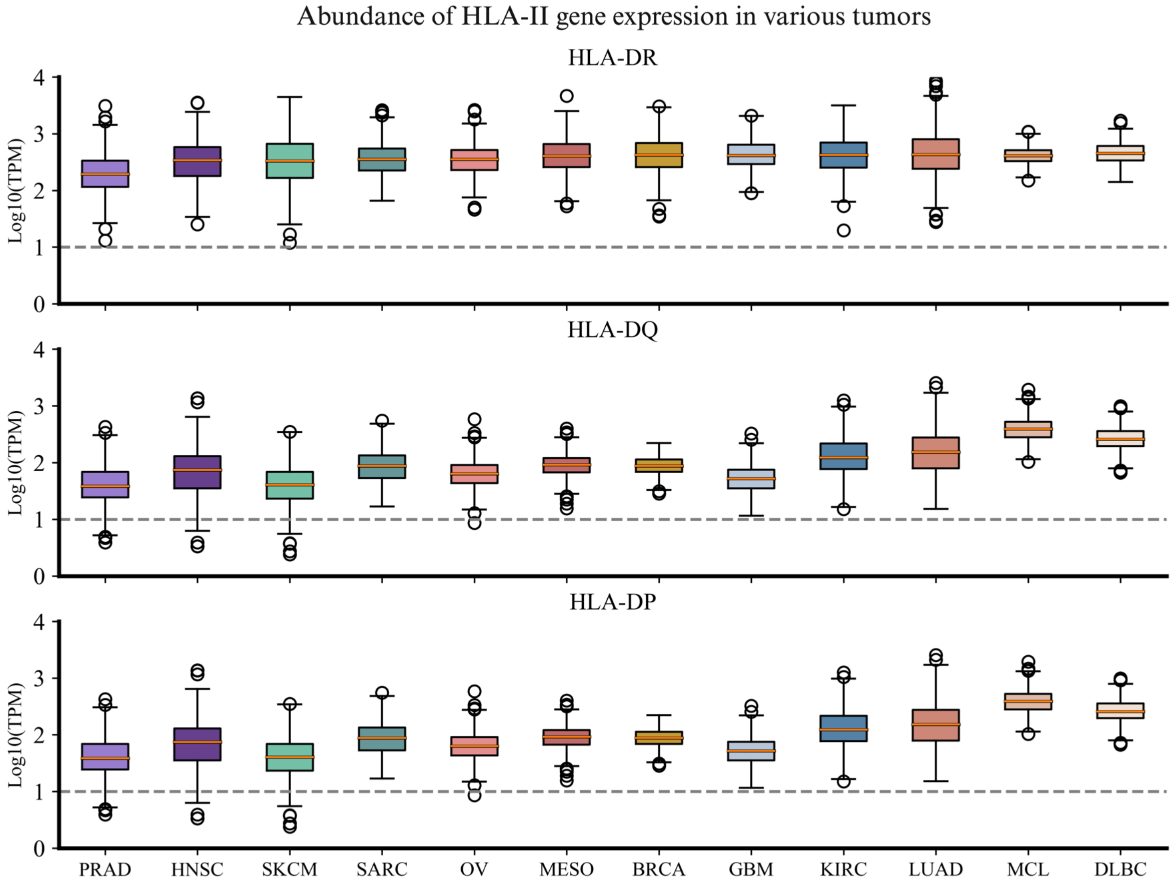

Supplementary Figure 2

Relationship between gene expression level and HLA-II ligands.

(a) Comparison of gene expression levels of presented and non-presented HLA-II in the EL dataset. Distributions of RNA-Seq estimated gene expression level from HLA-II ligands, whole genetic transcriptome and non-presented genes. (b) Ablation for filtering decoy peptide gene expression values on prediction performance of gene expression. When the lowly expressed genes were removed, the predictive capability decreased gradually. (c) Bulk RNA-Seq values of HLA-DR, HLA-DQ, and HLA-DP of 5077 TCGA tumor samples and 6 MCL tumor samples plotted as box plots. Top and bottom lines indicate 95% confidence interval, and the box indicates the first and third quartiles. PRAD: prostate adenocarcinoma, n=558; HNSC: head and neck squamous cell carcinoma, n=566; SKCM: skin cutaneous melanoma, n=473; SARC: sarcoma, n=265; OV: ovarian serous cystadenocarcinoma, n=430; MESO: mesothelioma, n=87; BRCA: breast invasive carcinoma, n=1256; GBM: glioblastoma multiforme, n=175; KIRC: kidney renal clear cell carcinoma, n=618; LUAD: lung adenocarcinoma, n=601; MCL: mantle cell lymphoma, n=8; DLBC: diffuse large B-cell lymphoma, n=48.

Supplementary Fig. 3

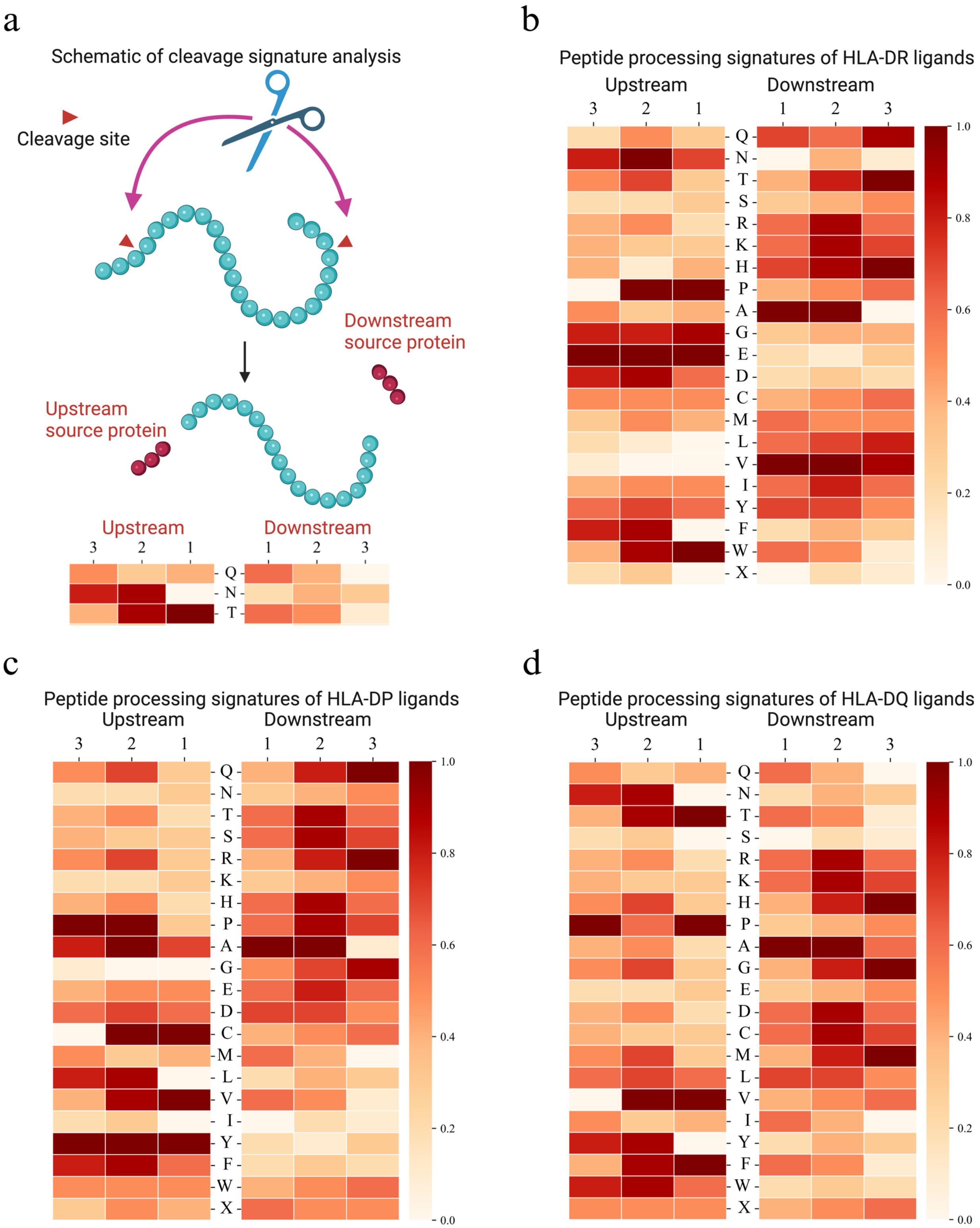

Supplementary Figure 3

Protease cleavage signature analysis across different HLA-II alleles.

(**a**) Schematic of cleavage signature analysis. The cleavage sites contained three aa upstream and three aa downstream of the HLA-II peptide N and C terminus in an N to C terminus order. (**b**) Peptide processing signatures of HLA-DR ligands. (**c**) Peptide processing signatures of HLA-DP ligands. (**d**) Peptide processing signatures of HLA-DQ ligands. The heatmaps are plotted to show the enrichment of the flanking residues of the peptide sequence.

Supplementary Fig. 4

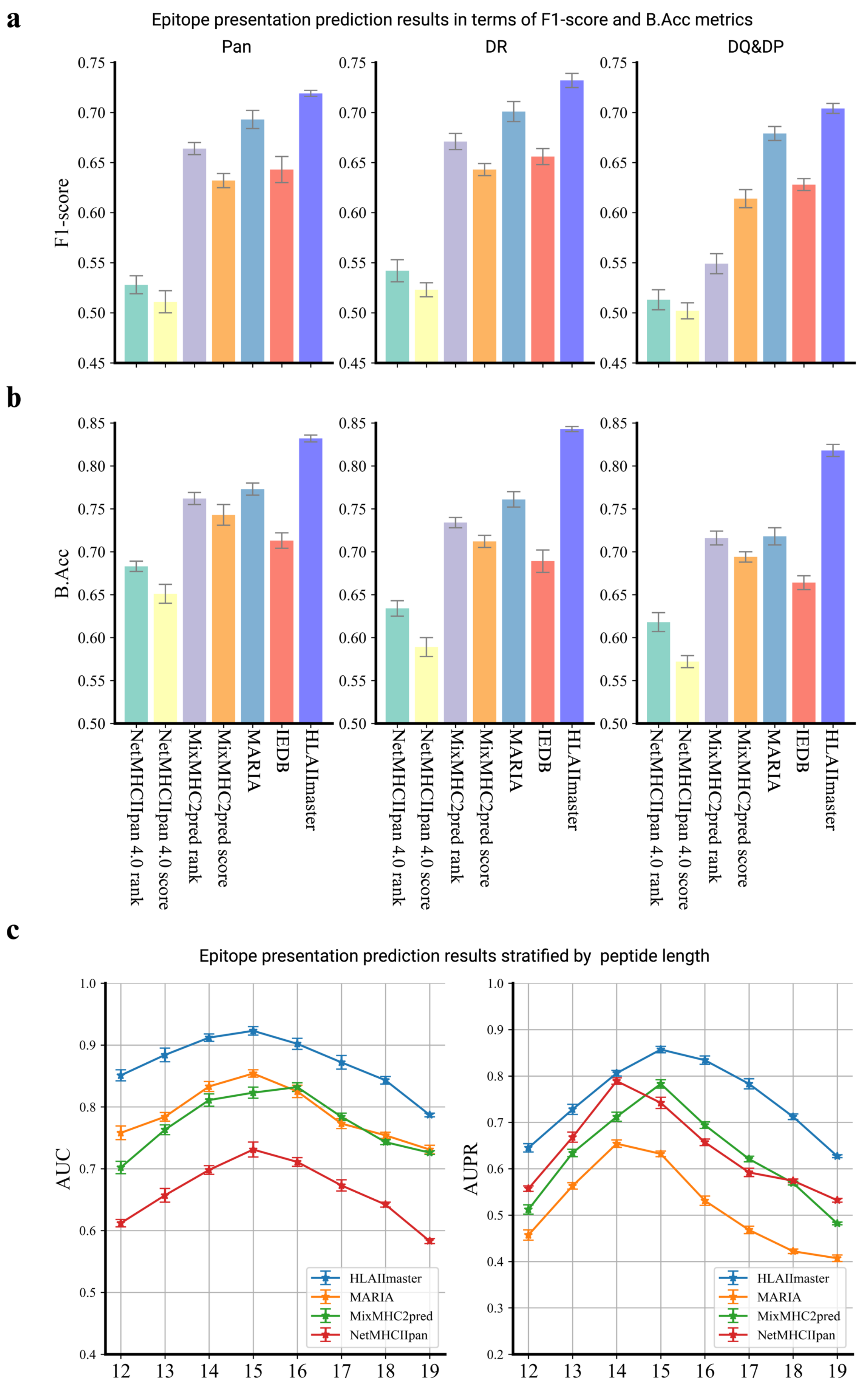

Supplementary Figure 4

Epitope presentation prediction results.

(**a**) F_1-scores results of HLAIImaster and advanced predictors around the pan-allele, the DR-specific, and the DQ&DP-specific models. (**b**) B.Acc results of HLAIImaster and advanced predictors around the pan-allele, the DR-specific, and the DQ&DP-specific models. According to the results, the proposed HLAIImaster is significantly improved in predicting HLA-II epitopes. (**c**) AUC and AUPR per peptide allele length with 95% CI by peptide stratification. As shown, the length of 15 often yields the best predictive score.

Supplementary Fig. 5

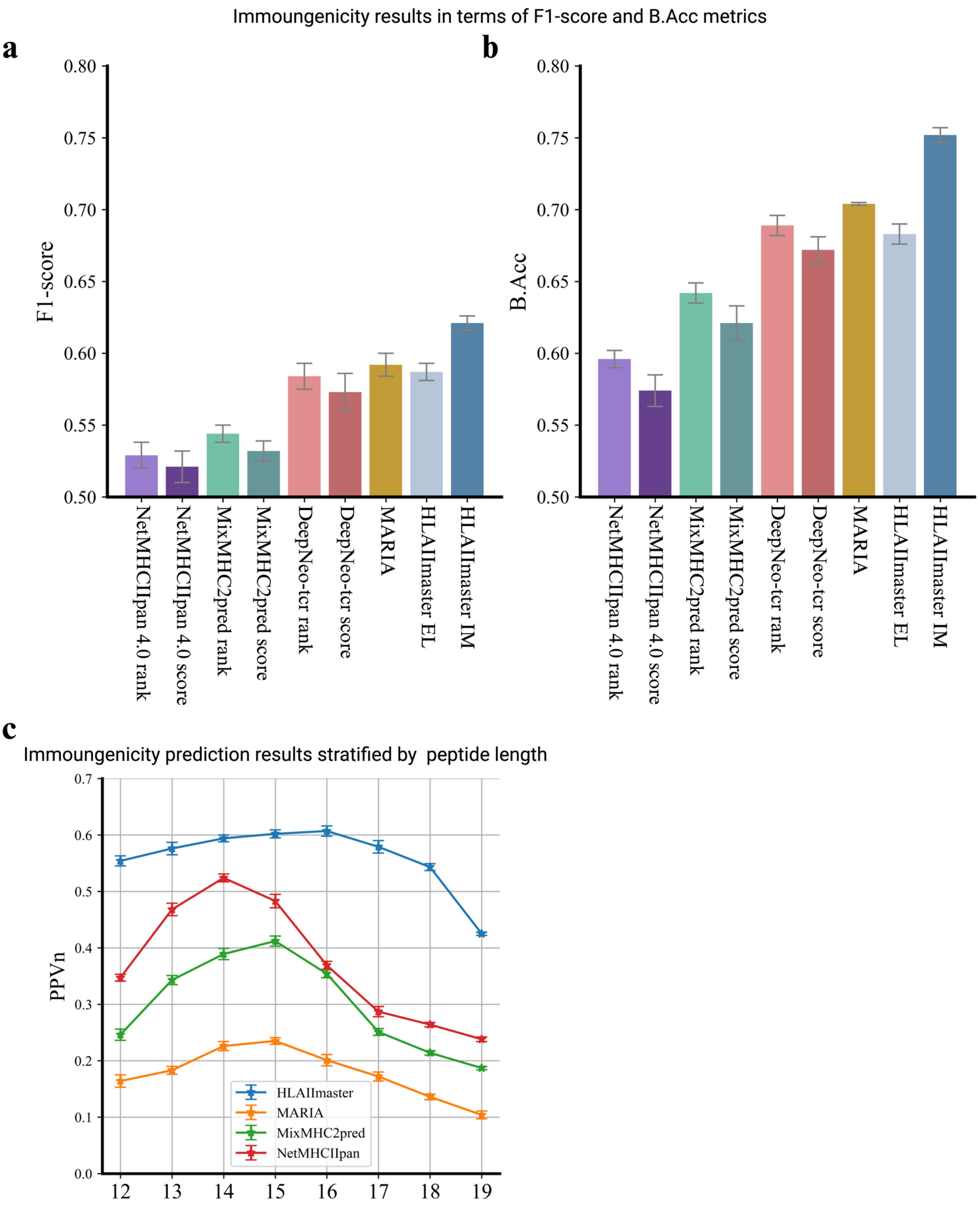

Supplementary Figure 5

Epitope immunogenicity prediction results.

(**a**) Comparison results of HLAIImaster and existing tools in terms of F_1-score. (**b**) Comparison results of HLAIImaster and existing tools in terms of B.Acc metrics. As shown, the performance of all models decreased sharply, but HLAIImaster is still superior to other tools. (**c**) PPVn per peptide allele length with 95% CI by peptide stratification.

Supplementary Fig. 6

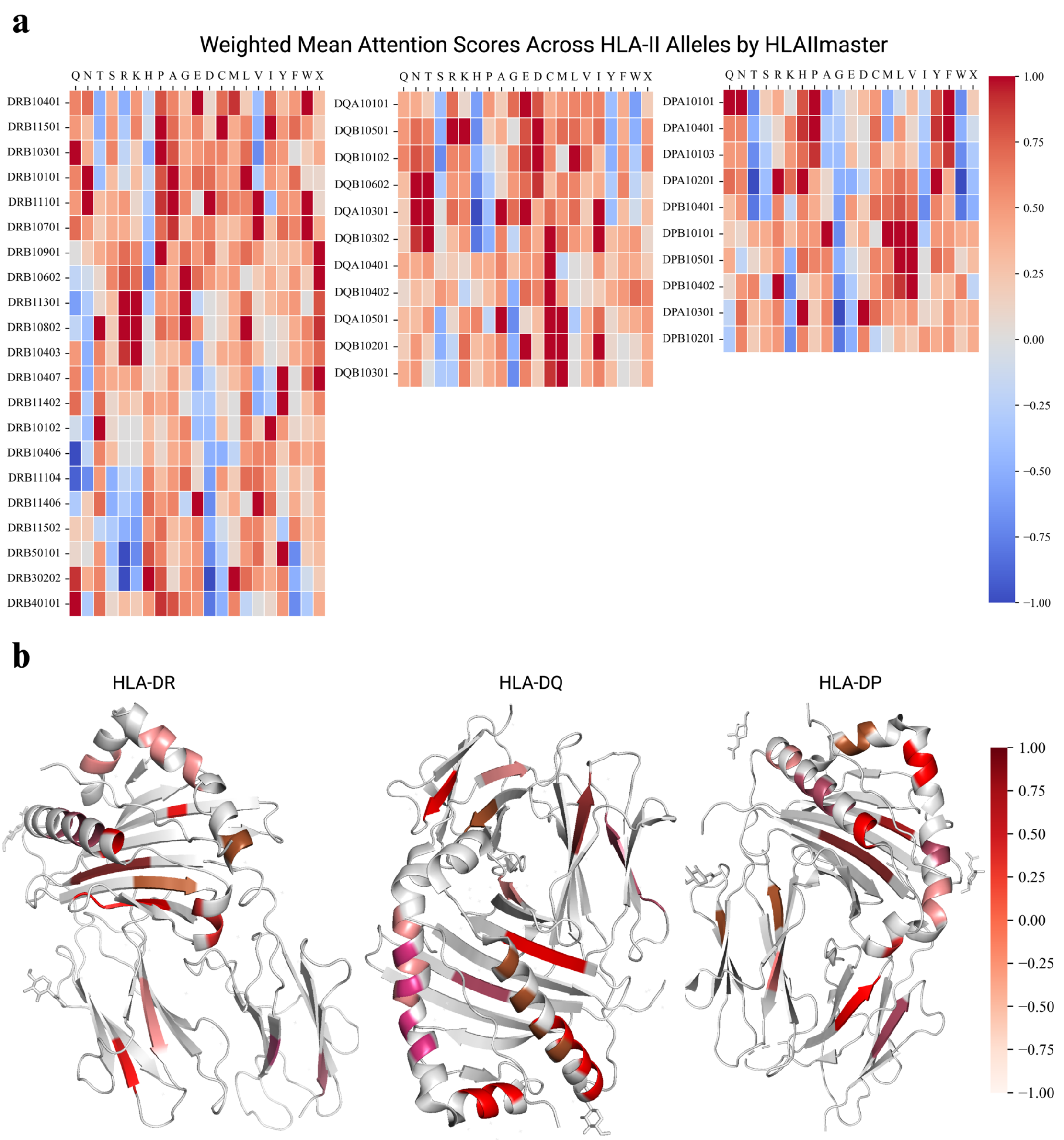

Supplementary Figure 6

Visualization of HLAIImaster attention to HLA encodings on the EL data.

(**a**)Heatmap visualization of the average attention value for each amino acid in the HLA pseudosequence on the EL dataset. The heatmap is stratified by MHC allele as rows, and separated by DR, DQ, DP loci. (**b**) Overlays of the Differences column from the EL dataset on the HLA molecule using Pymol. HLA protein structure models are generated using AlphaFold2.0.

Supplementary Fig. 7

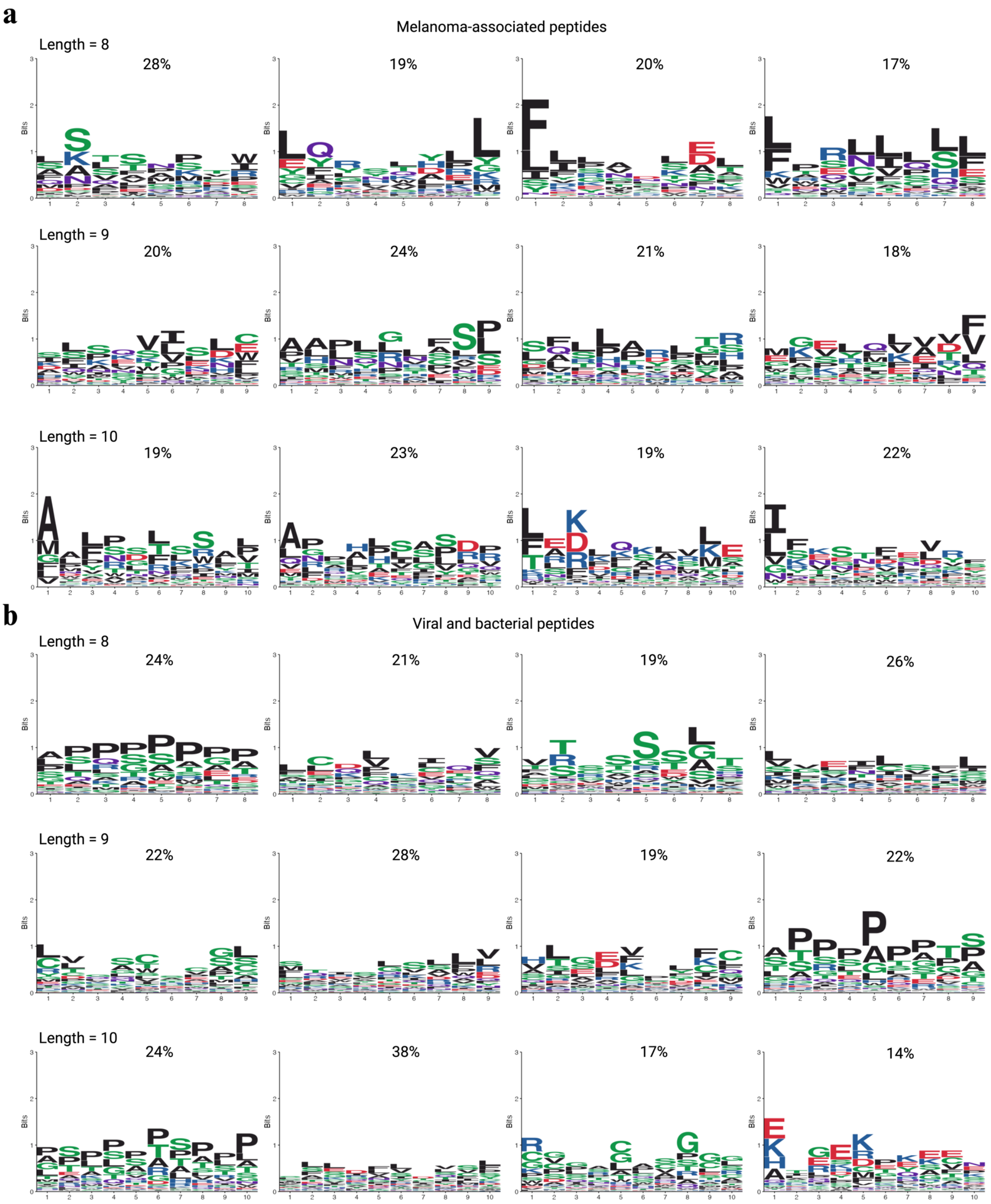

Supplementary Figure 7

The influence of binding core length for neoantigen identification in various tumors.

(**a**) Distribution of melanoma-associated peptide motifs stratified by binding core length. (**b**) Distribution of viral and bacterial peptide motifs stratified by binding core length.

Note 1

In this paper, we compared HLAIImaster with existing advanced predictors to evaluate the performance of HLA-II epitope identification, namely, NetMHCIIpan [1], MixMHC2pred [2], DeepNeo [3], MARIA [4] and IEDB tool [5].

NetMHCIIpan-4.0 predicts peptide binding to any MHC II molecule of known sequence using Artificial Neural Networks (ANNs). The network can predict for peptides of any length. The output of the model is a prediction score for the likelihood of a peptide to be naturally presented by and MHC II receptor of choice. The output also includes %rank score, which normalizes prediction score by comparing to prediction of a set of random peptides. Optionally, the model also outputs BA prediction and %rank scores.

MixMHC2pred is a powerful tool for robust prediction of HLA class II epitopes based on deep motif deconvolution of immunopeptidomes. This model also combines the features including pan-allele and specific-allele peptide N- and C- terminal motifs, and binding core offset preferences, providing more accurate and interpretable prediction for immunogenic epitope presentation.

DeepNeo is a CNN-based a pan-allele and multispecies model to identify immunogenic neoepitopes that can stimulate TCR responses and thus show signatures of negative selection under immune pressure.

MARIA is a deep neural network trained using the HLA-II ligands identified by MS-based antigen presentation profiling, along with empirical in vitro HLA binding measurements, and gene expression levels. It MARIA allows robust and more accurate HLA-II prediction, and that its performance gains are achieved by combining these improved training data with a new supervised machine learning model using a multimodal recurrent neural network (RNN).

Note 2

In this study, we applied the *Area Under the Receiver Operating Characteristic Curve* (AUC) and *the Precision-Recall Curve* (AUPR), F1-score and balanced accuracy metrics to evaluate the proposed deep learning model HLAIImaster. AUC is calculated by true positive rate (*TPR*, Y-axis) and false positive rate (*FPR*, X-axis).

$$TPR = \frac{TP}{TP + FN}, FPR = \frac{FP}{FP + TN}$$

where TP indicates the true positive samples, FN indicates the false negative samples, FP is the false positive samples and TN is the true negative samples.

AUPR is a metric to evaluate the model performance trained on an unbalanced dataset. Generally, AUPR is regarded as the area under the *Precision-Recall curve*, where *Precision* is the Y-axis and *Recall* is the X-axis.

$$Precision = \frac{TP}{TP + FP}, Recall = \frac{TP}{TP + FN}$$

F_1-Score is a statistical measure of the accuracy of binary classification models. It considers the precision and recall of the classification model at the same time. It is defined as follows:

$$F_1 - Score = 2 * \frac{Precision * Recall}{Precision + Recall}$$

B.Acc calculates the balanced accuracy, which avoids inflated performance estimated on imbalanced datasets. B.Acc is defined as the average between specificity and sensitivity at a certain threshold:

$$Sensitivity = \frac{TP}{TP + FN}, Specificity = \frac{TN}{FP + TN}$$

$$B.Acc = \frac{1}{2}(\ Sensitivity\ +\ Specificity)$$

Reference

[1] Reynisson B, Alvarez B, Paul S, et al. NetMHCpan-4.1 and NetMHCIIpan-4.0: improved predictions of MHC antigen presentation by concurrent motif deconvolution and integration of MS MHC eluted ligand data. *Nucleic Acids Res*. 2020;**48**(W1):W449-W454.

[2] Racle J, Michaux J, Rockinger GA, et al. Robust prediction of HLA class II epitopes by deep motif deconvolution of immunopeptidomes. *Nat Biotechnol*. 2019;**37**(11):1283-1286.

[3] Kim JY, Cha H, Kim K, et al. MHC II immunogenicity shapes the neoepitope landscape in human tumors. *Nat Genet*. 2023;**55**(2):221-231.

[4] Chen B, Khodadoust MS, Olsson N, et al. Predicting HLA class II antigen presentation through integrated deep learning. *Nat Biotechnol*. 2019;**37**(11):1332-1343.

[5] Vita R, Mahajan S, Overton JA, et al. The Immune Epitope Database (IEDB): 2018 update. Nucleic Acids Res. 2019;47(D1):D339-D343.
